# Kinetic effects of TiO_2_ fine particles and nanoparticles aggregates on the nanomechanical properties of human neutrophils assessed by force spectroscopy

**DOI:** 10.1186/2046-1682-6-11

**Published:** 2013-08-19

**Authors:** Everton Luis Santos da Rosa

**Affiliations:** 1 Laboratory of Biochemistry and Protein Chemistry, Department of Cell Biology, University of Brasilia, Campus Darcy Ribeiro, Brasilia 70910-900, Brazil

**Keywords:** Force spectroscopy, Neutrophil nanomechanics, Titanium dioxide microparticles, Titanium dioxide nanoparticles

## Abstract

**Background:**

Increasing applications of titanium dioxide (TiO_2_) fine particles (FPs) and nanoparticles (NPs) require coupled knowledge improvement concerning their biokinetic effects. Neutrophils are quickly recruited to titanium implantation areas. Neutrophils mechanical properties display a crucial role on cell physiology and immune responsive functions. Then, micro and nanomechanical characterization assessed by force spectroscopy (FS) technique has been largely applied in this field.

**Results:**

Scanning electron microscopy (SEM) images highlighted neutrophils morphological changes along TiO_2_ FPs and NPs aggregates exposure time (1, 5, and 30 min) compared to controls. FS approaches showed an increasing on attraction forces to TiO_2_ FPs and NPs treated neutrophils. This group depicted stronger stiffness features than controls just at 1 min of exposure. Treated neutrophils showed a tendency to increase adhesive properties after 1 and 5 min of exposure. These cells maintained comparatively higher elasticity behavior for a longer time possibly due to intense phagocytosis and cell stiffness opposing to the tip indentation. Neutrophils activation caused by FPs and NPs uptake could be related to increasing dissipated energy results.

**Conclusions:**

Mechanical modifications resulted from TiO_2_ FPs and NPs aggregates interaction with neutrophils showed increasing stiffness and also cell morphology alteration. Cells treatment by this metal FPs and NPs caused an increase in attractive forces. This event was mainly observed on the initial exposure times probably regarding to the interaction of neutrophils membrane and phagocytosis. Similar results were found to adhesion forces and dissipated energy outcomes. Treated cells presented comparatively higher elasticity behavior for a longer time. SEM images clearly suggested cell morphology alteration along time course probably related to activation, cytoskeleton rearrangement and phagocytosis. This scenario with increase in stiffness strongly suggests a direct relationship over neutrophil rolling, arrest, and transmigration. Scrutinizing these interactions represents an essential step to clarify the mechanisms involved on treatments containing micro and nanomaterials and their fates on the organisms.

## Background

Titanium dioxide (TiO_2_) has been widely used in automotive and aerospace industry, personal care, food, and even in pharmaceutical and biomedical products [1,2]. Increasingly, this metal and its alloys are used in the biomedical field for medicine and dentistry [3,4]. Pure titanium and some of its alloys are non-toxic and generally have been described as being biocompatible with human tissues [5]. Despite its biocompatibility, toxicological concerns are depicted in the literature related to ions, microparticles (MPs), fine particles and nanoparticles [6-10]. Despite being inert [11], titanium may promote inflammatory reactions by recruiting inflammatory cells [12], such as neutrophils.

Neutrophils represent the first line of innate defense, comprising about 50-70% of all human leukocytes [13]. They are quickly summoned to areas containing titanium micro or nanoparticles [14]. It is well-known that MPs and NPs can be internalized by phagocytic and even non-phagocytic cells [15-17], and can interact with cell membranes, subcellular organelles, proteins, and nucleic acids [18,19]. Neutrophils internalize TiO_2_ MPs and NPs, leading to a change in shape and motility; these events are modulated by cytoskeleton proteins, and enhance the production of superoxide anion, cytokines, and chemokines due to cell activation [20-23]. There are different pathways for the uptake of MPs and NPs: micro-sized particles are internalized via phagocytosis by monocytes, macrophages, and neutrophils [17], while NPs (ranging from 1 to 100 nm) seem to use other pathways in spite of the fact that, in some situations, NPs can aggregate or agglomerate, resulting in MPs behaviour, being an important factor in understanding cytotoxicity. However, the effect of the aggregate size of nanoparticles on cells is unclear [24]. Agglomeration (particles are linked by weak forces, eg van der Waals forces, capillary) and aggregation (particles held together by chemical bonds-strong forces) of TiO_2_ MPs and NPs are known mechanisms [25] and may occur on the surface of the cells membrane affecting communication with the external environment [26]. Furthermore it is known that the length and chemical nature of the aggregates of TiO_2_ are related to the toxicity [27]. In addition to phagocytosis, uptake pathways include clathrin-mediated endocytosis, caveolin-mediated endocytosis, macropinocytosis, and pathways independent of clathrin/caveolin. The passive pathway should also be considered for NPs uptake [28].

Neutrophil recruitment into areas with MPs or NPs proceeds in a cascade process. Rolling of neutrophils inside vessels is mediated by selectins (PSGL-1) and their counter-receptors (L and P selectins) and integrin-mediated (LFA-1, Mac-1) arrest [29]. These adhesion mechanisms activate different signaling pathways in the cell, leading to neutrophil extravasation, promoting cytoskeletal rearrangement, and inducing superoxide production and degranulation [30,31]. The behavior of neutrophils and other cells following contact with macro or micro-sized particles are different from following contact with NPs, due to specific surfaces, energy, and physicochemical properties [25,32,33]. When circulating neutrophils encounter a macro-sized TiO_2_ surface, the adhesion process starts, mediated by selectins, FcγIII receptor (CD16) and finally integrins (CD11b); cells are not activated to undergo a respiratory burst by TiO_2_ surfaces [34]. On the other hand, neutrophils phagocytose TiO_2_ NPs and MPs aggregates smaller than the cell [15,23]. Furthermore, TiO_2_ MPs and NPs may activate and induce alterations in cell morphology [20,35] and inhibit apoptosis (at 50–100 μg/mL). They also induce the production of several different cytokines/chemokines, mainly IL-8 and Gro-α [20]. Therefore, when this interaction occurs, MPs and NPs play a crucial role in cell physiology and mechanical properties. Moreover, the mechanical properties of cells are important when aiming to understand mechanisms such as adhesion, endothelial transmigration, and diapedesis [36].

Concerning the mechanical properties of cells, a variety of methods and techniques have been used to probe cells such as micropipette aspiration [37], magnetic twisting cytometry (MTC) [38], and atomic force microscopy (AFM). Conceptually, AFM is a type of scanning probe microscopy (SPM) and is perceived as a tool central to the goals of the burgeoning field known as nanotechnology [39]. It represents an effective tool to probe the viscoelastic properties of cells, to study the membrane and subcellular structures, and plays a key role in determining cell function in response to inflammatory stimuli [40,41]. AFM is mainly used for surface topography analysis by using attractive (short-range chemical, van der Waals and electrostatic forces) and repulsive interaction forces among a few atoms attached to the tip of a cantilever and the sample surface (nanoindentation). This technique has increasingly been applied for the characterization of single molecules under tensile or torsional load, cell-to-cell interactions, interactions of surfaces with molecules, and investigations into the mechanical properties of cells [42]. Further details concerning AFM are presented elsewhere [36,43,44]. The nanoindentation technique using force spectroscopy enables researchers to assess living or fixed neutrophils, in a passive or activated state, pointing out the micro-rheology of the entire cell surface (e.g. the cell body, lamellipodia, uropodia, and projections of the cytoskeleton), allowing the analysis of viscoelastic properties [45,46].

Force spectroscopy is the force-versus-distance measurement taken when using AFM in vertical mode only [47]. Cantilever deflection is recorded as it indents the cell, by reflecting a laser off the cantilever into a split photodiode. Hertzian mechanics gives a linear cell-based elastic model on AFM deflection data to determine cell elasticity, among other mechanical parameters [42,48]. Focusing on neutrophil rheology, Lee and coworkers stated that the neutrophil body is significantly stiffer than the regions closer to the leading edge, while leading edge and tail regions were mechanically indistinguishable [45]. On the other hand, Rosenbluth and coworkers, when comparing neutrophils, myeloid (HL60) and lymphoid (Jurkat) cells, reported that there is a significant cell type effect on stiffness. Neutrophils were significantly softer than HL60 cells and significantly stiffer than Jurkat cells [48].

Increasing applications of FPs and NPs in the dental and medical field (dental implants, orthopedic prostheses, and antineoplastic therapies) have led to concerns regarding the biokinetic effects of micro and nanomaterials and their fate *in vivo.* Therefore, the present study aimed to analyze some mechanical properties related to the effects of TiO_2_ FPs and NPs aggregates exposure on human neutrophils by a force spectroscopy approach over time (1, 5, and 30 min). Parameters like snap-in force, maximum load force, detachment force, Young’s modulus, and dissipated energy were depicted by approaching and retracting a non-functionalized tip over adherent cells on local nano-domains in Z-direction. Cell features like stiffness and elasticity were investigated.

## Methods

### Exposure times

The methodology of human neutrophils interaction with titanium dioxide FPs and NPs was adjusted and various exposure times were tested. Once the literature has few information concerning neutrophils interaction with TiO_2_ micro, submicron and nanoparticles by AFM, only the first hour were assessed by FS. However during experiments for this study, cellular analysis intervals tested above thirty minutes, resulted in locking the cantilever tip, by preventing to achieve the force curves measurements. Hence the exposition times used were 1, 5 and 30 minutes.

### TiO_2_ FPs and NPs characterization

Samples of 99.9% pure titanium oxide FPs and NPs (Sigma-Aldrich, TiO_2_ reference number 14027, titanium (IV) oxide puriss) were immersed in 1 mL of Milli-Q water using a 5 mL sterilized disposable syringe. Particle hydrodynamic diameter was analyzed by dynamic light scattering (DLS) in triplicate using a Nanotrac U2131I (Microtec Inc., USA, at Instrutécnica, SP). Moreover, TiO_2_ FPs and NPs were sized by Transmission Electron Microscopy (TEM) analysis of the dry powder (JEM- 2011, JEOL Ltd.) and also by AFM. Particles were sterilized in an autoclave at 121°C for 20 min [49]. Suspensions containing 200 μg/mL TiO_2_ FPs and NPs were submitted to ultrasound bath for 30 min before use, aiming to avoid particles aggregation.

### Human neutrophil preparation

Neutrophils were isolated from human whole blood using a density gradient technique as described in a previous study [50]. Briefly, 12 mL of peripheral blood were drawn from three healthy individuals with sterile heparinized syringes and not pooled. Collected blood was added to a Percoll density gradient (60%/70%) and centrifuged. After centrifugation, polymorphonuclear cells were harvested from the interface of the two gradients using a Pasteur pipette and re-suspended in 500 μL of Ca-/Mg-free blood buffer in Hank’s Balanced Salt Solution (HBSS; Sigma-Aldrich, St. Louis, Missouri Sigma-Aldrich Corp.). The suspension containing 7 × 10^5^ cells was divided into six aliquots: three were incubated (1, 5, and 30 min) with 20 μL of the TiO_2_ FPs and NPs suspension at 37°C and three were kept as controls (1, 5, and 30 min). After incubation, cells were fixed with 100 μL of Karnovsky solution (12% paraformaldehyde + 8% glutaraldehyde + 0.2 M cacodylate buffer). Once fixed, cells were washed with PBS for 5 min and centrifuged for 2 minutes, three times (4500 *g* at 4°C). Each cells aliquot (1, 5, and 30 min) was then placed on separated pre-sterilized circular coverslip. For force spectroscopy measurements, only well-adhered cells were selected.

### Ethics and consent statement

All the experiments were performed with the approval of the Research Ethics Committee of Medical Faculty, University of Brasilia (registration number: CEP-CFM-45/2010), in compliance with the Helsinki Declaration. Likewise, adult participants in the study (all human subjects) provided informed consent for blood donation.

### Scanning electron microscopy (SEM) analyses

Fixed neutrophils were buffered in 0.1 M sodium cacodylate buffer and 0.1 M osmium tetroxide for 1 h, dehydrated and sputter-coated with gold. A SEM microscope (Jeol 840A operating at an accelerating voltage of 20 kV was used to depict possible morphological changes in neutrophils.

### Force spectroscopy analyses

Commercial AFM equipment (SPM-9600, Shimadzu, Japan) operated in air contact mode was used to analyze surface interaction forces between the tip atoms and cell membranes. Circular coverslips (10 mm) containing adhered neutrophils of each sample were fixed on a metallic support using a double-sided adhesive tape. A pyramidal silicon nitride tip (curvature radius < 20 nm) integrated with a cantilever with length of 200 μm (spring constant 0.15 N/m and approximately 24 kHz resonance frequency) was used in this technique. The acquisition rate was 1 Hz with 20 V of amplitude and operating point 3 V. At each incubation time point (1, 5, and 30 min) 10 cells from each group (control and TiO_2_) were analyzed and five force curves per cell were measured, resulting in 300 events, taking into account the control and experimental groups.

### Data analysis

SPIP v. 5.1.5 was used to force curve analysis set at 23°C and 0.15 N/m spring constant, cone indentation (Sneddon), with five fitting points and the approach curve as the baseline correction curve. FS data were further analyzed using Origin 8.6 software by ANOVA and the Tukey test (P < 0.05).

## Results and discussion

Particle size is a primary parameter when considering the effect of MPs, FPs and NPs in cellular interactions. According to Watari et al. [35], increasing the specific surface area usually implies a micro or nanosizing effect, which enhances chemical reactivity and consequently leads to serious toxicity for soluble and suspended materials. Watari et al. [51] considered particle size biointeractive below cell size, namely approximately 10 μm or smaller. These authors also point out critical sizes related bioreactivity; the micro or nanomaterial critical size means that, below 200 nm, a particle becomes invasive to the organism’s structures, leading to a lack of recognition of this material by the immune system [35,51]. Furthermore, TiO_2_ particles and nanoparticles aggregation is related to understanding cytotoxicity. It is known that the surface area, length and chemical nature of the TiO_2_ MPs and NPs aggregates are related to the cell toxicity [24,27]. It has been shown that nanoparticles are able to promote a more pronounced toxicity effect than microparticles [52]. Once the MPs and NPs have great potential for agglomeration and aggregation it is unlikely to occur cellular exposure to isolated form of this particles [53]. Okuda-Shimazaki et al. [24] demonstrated large (596 nm) TiO_2_ aggregates showed a larger effect on cell viability and gene expression when compared with the small aggregates (166 nm). This suggests that particle aggregate size is related to cellular effects. Bermudez et al. [54] exposed rats to inhalation of TiO_2_ NPs (21 nm) for the evaluation of pulmonary toxicity. Even with the aerosols formed was possible to detect the presence of particles aggregates with 1.37 μm, which were able to promote cytotoxicity.

**Figure 1:**
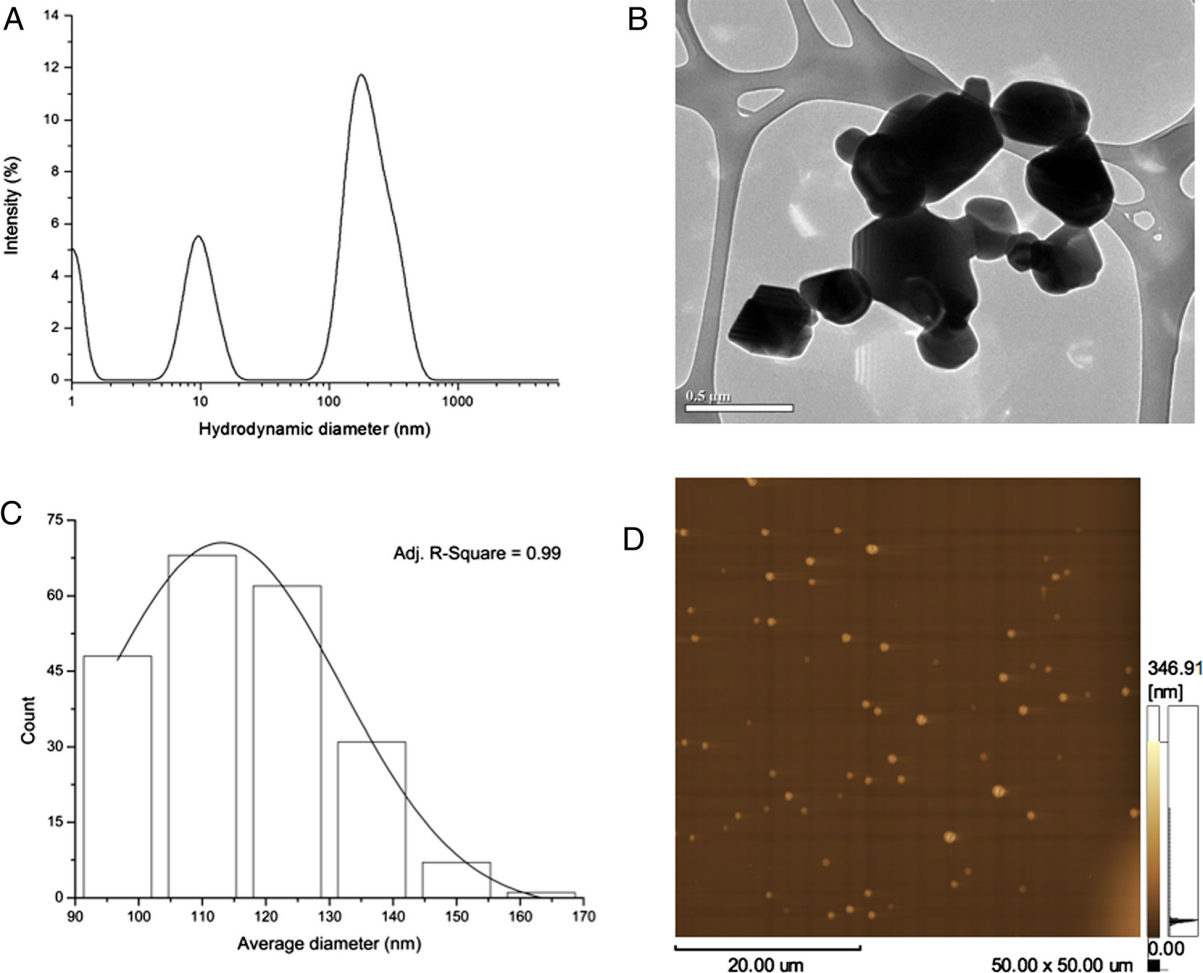
**Characterization of TiO**_**2 **_**FPs and NPs by DLS, TEM and AFM.** Hydrodynamic diameter distributions of TiO_2_ particles measured by dynamic light scattering (DLS) analysis **(A)**. TiO_2_ fine particles and nanoparticles characterized by TEM **(B)**. Fine particles and nanoparticles (aggregates) average diameter by AFM **(C)**. AFM image of TiO_2_ aggregates **(D)**.

Figure 1 shows the TiO_2_ FPs and NPs aggregates hydrodynamic diameter distributions (A), where approximately 67% of the particles were about 180 nm in hydrodynamic diameter (see Additional file 1: Table S1- Peak Summary). The TEM image showed TiO_2_ aggregates with size from NPs up to FPs and MPs (B). Also AFM demonstrated an average diameter below 170 nm with a peak at 115 nm (C). AFM image of TiO_2_ aggregates (D).

**Figure 2:**
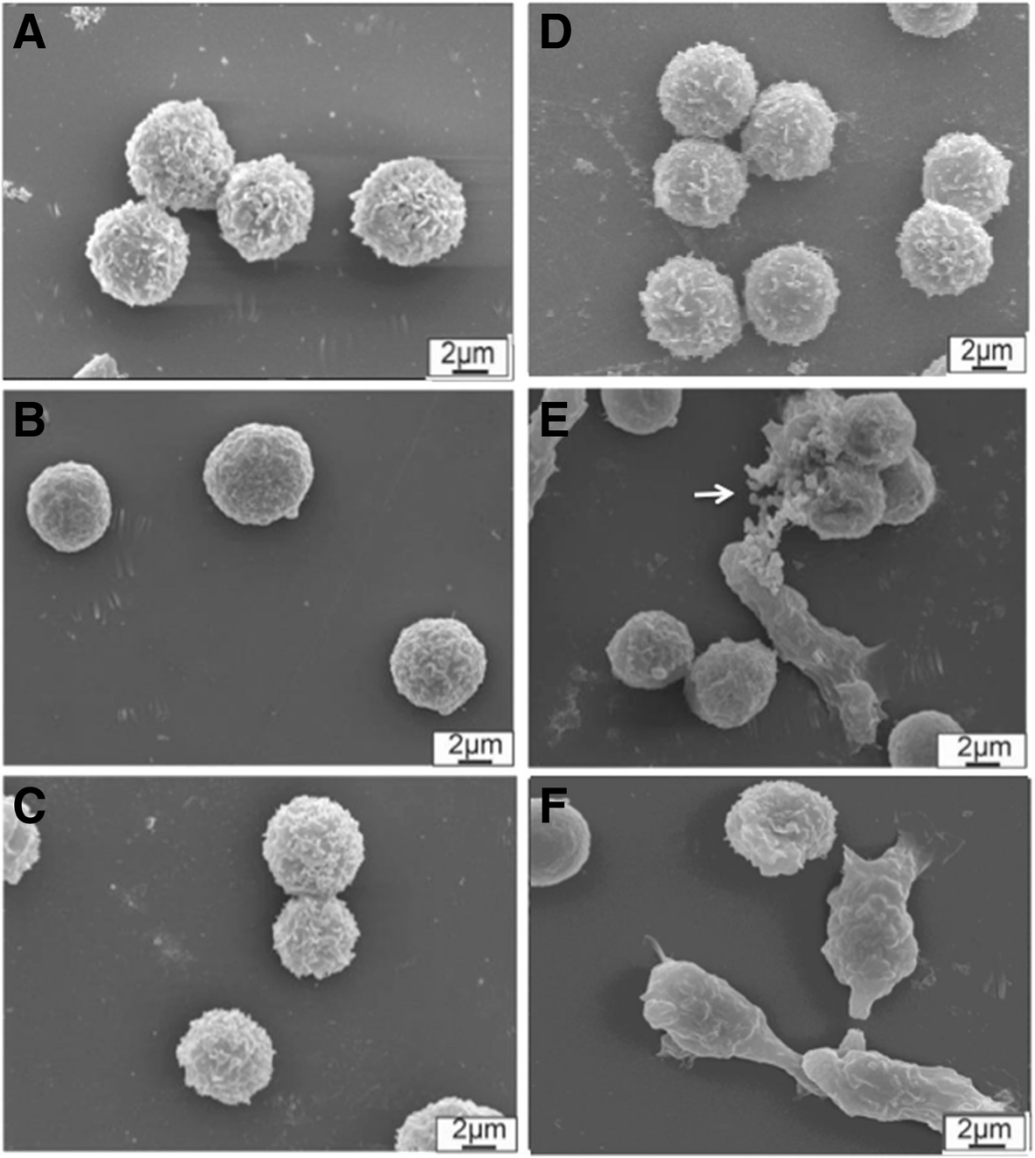
**SEM images of controls and TiO2 FPs and NPs aggregates exposed neutrophils.** Images of untreated neutrophils or control **(A**, **B**, and **C)** and TiO_2_ fine particles and nanoparticles clusters- exposed neutrophils **(D**, **E**, and **F)** obtained by scanning electron microscopy (SEM) analyses. **A** and **D** refer to cells collected after 1 min of exposure, **B** and **E** after 5 min, and **C** and **F** after 30 min.

Figure 2 shows SEM images comparing non-exposed neutrophils (A, B, and C) and TiO_2_ particles aggregates exposed neutrophils (D, E, and F) after 1 min (A and D), 5 min (B and E), and 30 min (C and F), respectively. TiO_2_ FPs and NPs aggregates are seen close to neutrophils (Figure 2E, white arrow).

It is possible to observe neutrophil morphological changes with increasing exposure time due to the influence of TiO_2_ aggregates. Neutrophils left the quiescent state (Figure 2A,B, and C) and transitioned to an activated form, extending their pseudopodia in order to phagocytose TiO_2_ aggregates (Figure 2D,E, and F). Several other studies [15,23,55] have demonstrated similar morphological alterations resulting from TiO_2_ FPs and NPs treatment, characterized by increasing cytotoxic biomarker expression (lactate dehydrogenase, superoxide anion, and proinflammatory cytokines), and inversely related to particle size. However, there is a lack of knowledge concerning the nanomechanical modifications resulting from TiO_2_ FPs and NPs aggregates exposure. The next step in this study was to evaluate the nanomechanical effects caused by the presence of TiO_2_ aggregates on the neutrophil surface at the same time points, using the FS technique.

**Figure 3:**
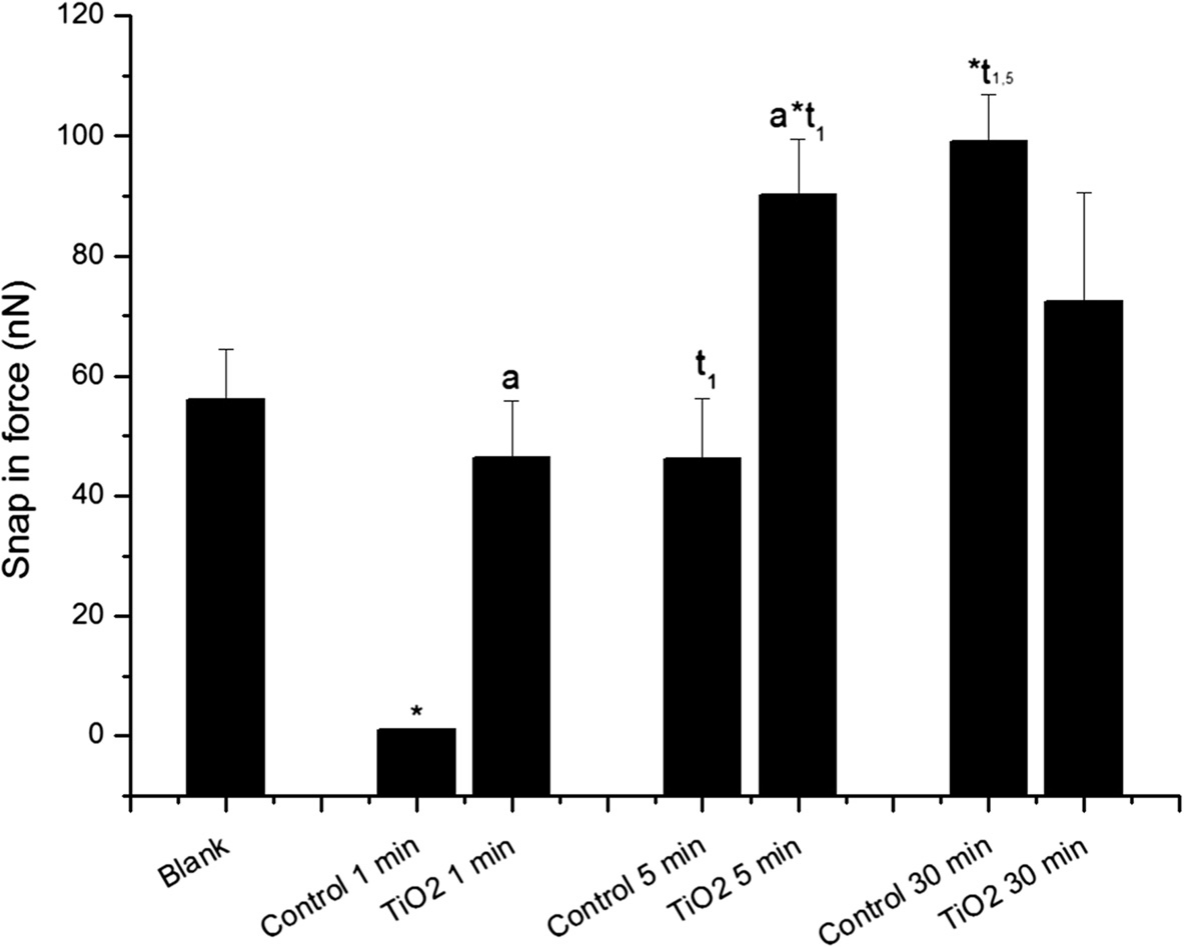
**Snap-in force calculated from force spectroscopy curves measured by AFM operating in contact mode.** “*Blank*” refers to bare circular silica coverslip (10 mm) surface analyses, and “*TiO*_*2*_*1, 5, and 30 min*” refer to force curve analyses obtained from neutrophils adhered to circular silica coverslips (10 mm) and exposed to TiO_2_ fine particles and nanoparticles aggregates for 1, 5, and 30 min. “*Control 1, 5, and 30 min*” refer to neutrophils adhered to circular silica coverslips (10 mm) without any TiO_2_ fine particles and nanoparticle treatment, collected at the same time as treated samples (1, 5, and 30 min). The (*) symbol indicates a statistically significant difference between “*Blank*” and samples (P < 0.05), and (a) indicates a statistically significant difference to the respective control, “t1” to “*Control 1 min*”, “at_1_” to “*TiO*_*2*_*1 min*” and “t_1,5_ to “*Control 1 min*” and “*Control 5 min*” (P < 0.05).

The FS results were derived from force-distance curves consisting of an AFM tip approximation and detachment from the sample surface. This event was monitored by an optical laser signal, which reflects from the upper reflective surface of the cantilever. Attractive, repulsive, and/or adhesive forces can manifest while this process occurs. The first event related to tip approximation involves attractive forces (such as van der Waals forces and capillarity interactions) and it is known as snap-in, whereupon the tip “jumps” to establish close contact with the sample surface. Figure 3 shows statistically significant differences in snap-in measurements between control and TiO_2_ aggregates treated neutrophils after 1 and 5 min of exposure. The treatments caused an increase in snap-in force measurements when compared to the control. These results could be related to morphological changes caused by neutrophil stimulation, as observed in Figure 2. The control cells showed a time-dependent increase in snap-in force values from 1 to 30 min, while TiO_2_ aggregates exposed cells showed increased force values from 1 to 5 min. Moreover, statistically significant differences (P < 0.05) were also found between aggregates exposed cells and their respective controls for the 1 and 5 min incubation times, but not at 30 min (Figure 3). These data may demonstrate that attractive forces like van der Waals and electrostatic forces on the cell membrane are more intense with longer incubation times. The interaction between FPs and NPs aggregates and the neutrophil membrane and uptake by phagocytosis increase the snap-in forces. Such forces seem to plateau at some point near 30 min, where the interaction with particles aggregates no longer enhances the values. Additional experiments with longer exposure times could demonstrate the behaviour of snap in forces along time.

Variations in sample surface mechanical properties such as viscoelasticity and adhesion forces, as well as fixation solutions, also influence cantilever oscillations [56,57]. On the other hand, one should consider that FPs and NP interference with neutrophil membranes is related to surrounded or attached TiO_2_ particles. This event could promote interactions with the tip, considering the ability of FPs and NPs to aggregate into numerous large clusters, as seen in Figure 2D and E [58]. For example, a study Guduru [56] demonstrated that bare polylactic-co-glycolic acid (b-PLGA) NPs interacted with ovarian cancer cells (SKOV-3) and after 24 h of incubation, and most of the particles remained on the cell surface. NPs in close contact with the membrane surface may promote a lack of communication with the external environment [26].

**Figure 4:**
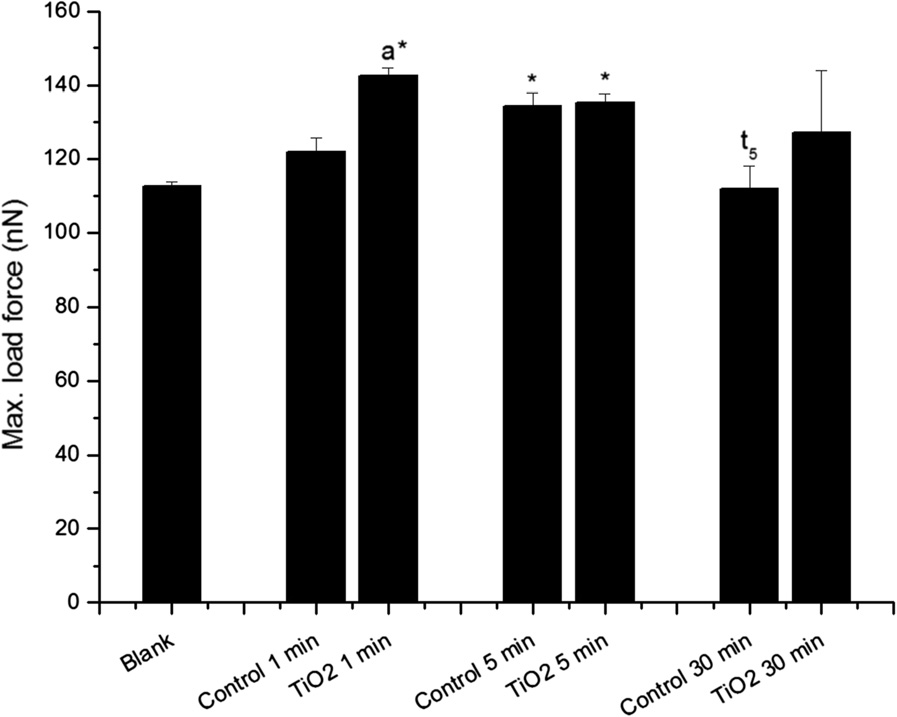
**Maximum load force calculated from force spectroscopy curves measured by AFM operating in contact mode.** “*Blank*” refers to bare circular silica coverslip (10 mm) surface analyses, and “*TiO*_*2*_*1, 5, and 30 min*” refer to force curve analyses obtained from fixed neutrophils adhered to circular silica coverslips (10 mm) and exposed to TiO_2_ fine particles and nanoparticles aggregates for 1, 5, and 30 min. “*Control 1*, *5*, and *30 min*” refer to fixed neutrophils adhered to circular silica coverslips (10 mm) without any TiO_2_ fine particles and nanoparticles treatment, collected at the same time as treated samples (1, 5, and 30 min). The (*) symbol indicates a statistically significant difference between “*Blank*” and samples (P < 0.05), and (a) indicates a statistically significant difference to the respective control and t5 to “*Control 5 min*” (p < 0.05).

The next event occurring in a force curve experiment is associated with the maximum force applied that relates to sample surface micro and nanostiffness. Maximum loading force measurements comparing control and TiO_2_ aggregates treated neutrophils showed statistically significant differences, but only at 1 min of exposure (Figure 4). Interestingly, mechanical assays showed that activated neutrophils were over two-fold stiffer than quiescent cells [59]. Moreover, under load, the membrane deformed in a non-linear elastic manner and demonstrated binary behavior of stiff and slack conditions [60]. Control cells at 30 min had a statistically significant difference to the respective control at 5 min (P < 0.05), suggesting a possible increase in membrane softness in quiescent neutrophils as time passes. Furthermore, when compared to control cells, TiO_2_ treated neutrophils at 1 min depicted stronger forces, which could represent increased cell stiffness due to phagocytosis or TiO_2_ aggregates adhesion to the cell membrane. It must be considered that, during phagocytosis, activated neutrophils might undergo increasing membrane cortical tension together with progressive cell surface and cytoskeletal structure hardening [61].

**Figure 5:**
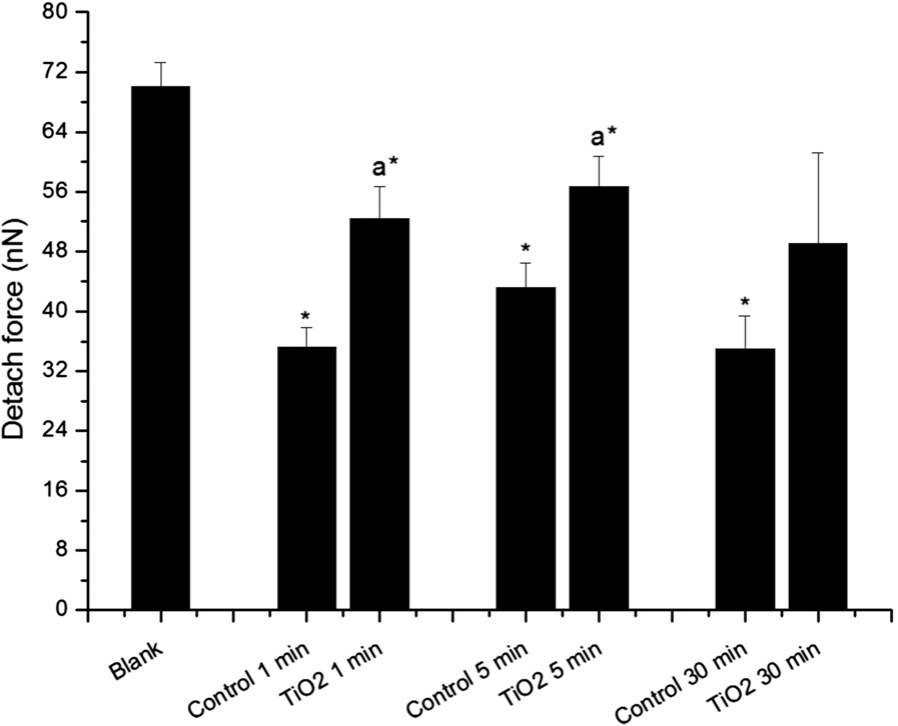
**Detach force calculated from force spectroscopy curves measured by AFM operating in contact mode.** “*Blank*” refers to bare circular silica coverslip (10 mm) surface analyses, and “*TiO*_*2*_*1, 5, and 30 min*” refer to force curve analyses obtained from fixed neutrophils adhered to circular silica coverslips (10 mm) and exposed to TiO_2_ fine particles and nanoparticles aggregates for 1, 5, and 30 min. “*Control 1, 5, and 30 min*” refer to fixed neutrophils adhered to circular silica coverslips (10 mm) without any TiO_2_ fine particles and nanoparticles treatment, collected at the same time as treated samples (1, 5, and 30 min). The (*) symbol indicates a statistically significant difference between “*Blank*” and samples (P < 0.05), and (a) indicates a statistically significant difference to the respective control (P < 0.05).

Detachment force measurement represents a relationship with adhesion forces (i.e. hydrogen bonds, ionic attractions, van der Waals forces, hydrophobic interactions, and/or chemical adhesion) [62]. This FS parameter occurs last on a retraction curve, corresponding to the point where the tip and surface lose contact, and it is related to adhesive interactions between them [63]. Figure 5 shows statistically significant differences (P < 0.05) between control and TiO_2_ treated neutrophils after 1 and 5 min of exposure, evidencing a tendency to increase this mechanical feature after stimulation. Once stimulated, cells acquire a polarized morphology, with F-actin polymerization in which integrins connect the cytoskeleton with the extracellular matrix [56]. Control cells at 1, 5, and 30 min demonstrated a statistically significant difference from the silica coverslip (P < 0.05). Stronger adhesion forces were seen with the glass than with quiescent neutrophil membrane surfaces, which showed that the adhesion forces were not due to the cell support. Similarly, TiO_2_ FPs and NPs aggregates treated neutrophils at 1 and 5 min had lower detachment forces than the silica coverslip.

**Figure 6:**
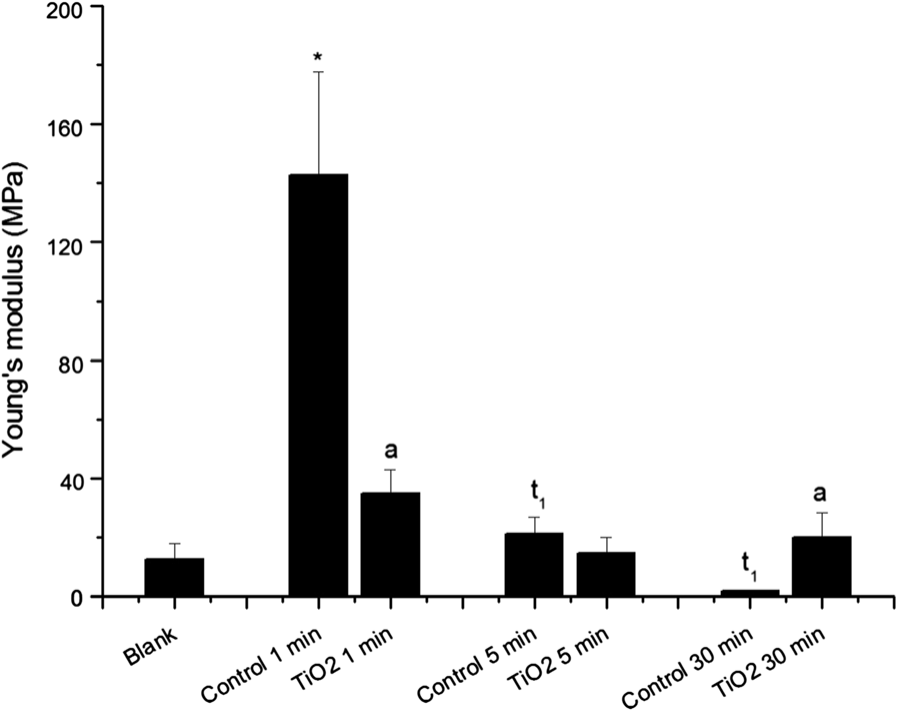
**Young’s modulus calculated from force spectroscopy analysis measured by AFM operating in contact mode.** “*Blank*” refers to bare circular silica coverslip (10 mm) surface analyses, and “*TiO*_*2*_*1, 5, and 30 min*” refer to force curve analyses obtained from fixed neutrophils adhered to circular silica coverslips (10 mm) and exposed to TiO_2_ nanoparticles for 1, 5, and 30 min. “*Control 1, 5, and 30 min*” refer to fixed neutrophils adhered to circular silica coverslip (10 mm) without any TiO_2_ fine particles and nanoparticles treatment, collected at the same time as treated samples (1, 5, and 30 min). The (*) symbol indicates a statistically significant difference between “*Blank*” and samples (P < 0.05), and (a) indicates a statistically significant difference to the respective control and t1 to “*Control 1 min*” (P < 0.05).

Figure 6 shows a comparison of Young’s modulus measurements between control and TiO_2_ aggregates treated neutrophils. Generically, this parameter relates to a sample’s elasticity/mechanical properties. The Young’s modulus of treated samples was smaller than that observed for control cells, demonstrating a comparatively higher elastic behavior over a longer period of time. Control and TiO_2_ aggregates treated neutrophils at 1 min demonstrated significant differences (P < 0.05). At the first time point, quiescent cells were much stiffer than treated cells. At 5 min, no statistically significant differences were seen. This may be related to cell activation and assembly of the cytoskeleton in the TiO_2_ group, thus increasing cell hardness. Furthermore, a remarkable transition occurred at 30 min. Treated neutrophils showed a statistically significant difference (P < 0.05), where it could be seen that intense phagocytosis and cell stiffening opposed the indentation of the cantilever tip. Similar results were found by Roca-Cusachs and coworkers who described adhered/activated neutrophils as being stiffer than quiescent cells [59].

Cell image analyses at 5 and 30 min (Figure 2E and F) showed remarkable changes in morphology, representing possible stimulation, F-actin rearrangement and migration to mediate TiO_2_ FPs and NPs aggregates phagocytosis.

Figure 6 shows that quiescent cells and control cells had a strict time-dependent decay pattern. Control cells at 5 and 30 min showed statistically significant differences to 1 min (P < 0.05). These features points to a quiescent cell elasticity modulus that decreased throughout the time course [36]. By transposing into a biological environment, cell deformation implies high stiffness, favoring neutrophil arrest at the capillary wall and subsequent adhesion. On the other hand, cell softening and slow deformation would facilitate the neutrophil transmigration progress [64].

**Figure 7:**
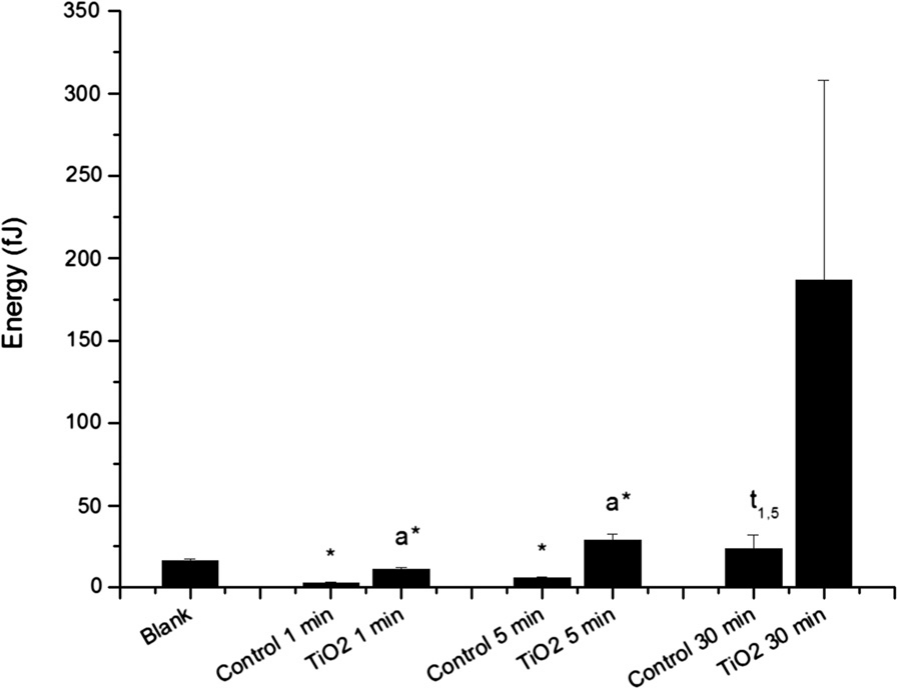
**Dissipated energy calculated from force spectroscopy analysis measured by AFM operating in contact mode.** “*Blank*” refers to bare circular silica coverslip (10 mm) surface analyses, and “*TiO*_*2*_*1, 5, and 30 min*” refer to force curve analyses obtained from fixed neutrophils adhered to circular silica coverslips (10 mm) and exposed to TiO_2_ nanoparticles for 1, 5 and 30 min. “*Control 1, 5, and 30 min*” refer to fixed neutrophils adhered to circular silica coverslips (10 mm) without any TiO_2_ fine particles and nanoparticles treatment, collected at the same time as treated samples (1, 5, and 30 min). The (*) symbol indicates a statistically significant difference between “*Blank*” and samples (P < 0.05), and (a) indicates a statistically significant difference to the respective control and t_1,5_ to “*Control 1 min*” and “*Control 5 min*” (P < 0.05).

Finally, Figure 7 shows cells the dissipated energy behavior over time. Dissipated energy results from tip and sample interactions as a function of either the gap distance or applied bias [65]. Vorden et al. [66] described non-conservative forces as being strongly dependent on these interactions. Energy dissipation can be related to sample viscoelastic properties [67]. A major contributor to energy dissipation is related to AFM tip/cell membrane contact with the lipid bilayer and cytoskeleton. Neutrophils do not have a homogeneous surface due to their complex inner composition, including a nucleus (thicker cell region by ~5 μm), cytoplasm, and cytoskeleton [57]; thus, different force indentation measurements are expected [44]. The cytoskeleton is known to consist of actin filaments, microtubules, and intermediate filaments with many accessory proteins [68]. In response to TiO_2_ stimulation, neutrophils remodel the cytoskeleton and increase cell rigidity, probably contributing to the non-linear and time-dependent mechanical properties. Dissipated energy measurements of TiO_2_ FPs and NPs aggregates treated neutrophils showed a strikingly increased tendency over time. A statistically significant difference (P < 0.05) between control and TiO_2_ aggregates treated neutrophils after 1 and 5 min of exposure was found, which may correspond to the activation of neutrophils and account for the energy dissipation enhancement as MPs and NPs uptake by cells occurred and rigidity increased. Focusing on the controls, the statistical analysis showed significant differences (P < 0.05) between 30 min and both 1 and 5 min control cells. It is possible that quiescent neutrophils could experience some degree of cytoskeletal organization and therefore increasing rigidity along the time course, which would increase the dissipation of energy when the tip is in close contact with the sample.

## Conclusions

The present study evaluated the effects of TiO_2_ FPs and NPs aggregates on neutrophil nanomechanical properties along a time course. Scanning electron microscopy was used to investigate cell morphological changes. Aiming to correlate the morphological and nanomechanical outcomes, force spectroscopy analyses were performed. To assess the attractive and adhesive interaction patterns between AFM tip and cell surface atoms, parameters like snap-in, detachment, and maximum load force were evaluated, as well as the elasticity modulus (Young’s modulus) and dissipated energy. Cells treated with TiO_2_ FPs and NPs aggregates showed an increase in attractive force measurements at initial exposure times compared with control cells, which may be related to the AFM tip interaction with activated neutrophils as well as with FPs and NPs aggregates on the cell membrane. Similar results were found for adhesion forces and dissipated energy measurements. Treated neutrophils showed stronger stiffness features than controls at 1 min of exposure and presented comparatively higher elastic behavior for a longer period of time. These results point to increased cellular nanostiffness in response to TiO_2_ FPs and NP aggregates treatment. This suggests an intrinsic connection concerning neutrophil morphological alterations with important biological events, such as adhesion and transmigration. A more complete understanding of these interactions will be an indispensable step to elucidate the mechanisms involved in treatments containing nanomaterials, how they interfere with modulation pathways, and the effect on organisms.

## Abbreviations

TiO2: Titanium dioxide; MPs: Microparticles; FPs: Fine particles; NPs: Nanoparticles; SPM: Scanning probe microscopy; AFM: Atomic force microscopy; SEM: Scanning electron microscopy; PSGL-1: P-selectin glycoprotein ligand-1; LFA-1: Lymphocyte function-associated antigen 1; Mac-1: Macrophage-1 antigen; FcγIII receptor: Low affinity immunoglobulin gamma Fc region receptor III; CD11b: Cluster of differentiation 11b; CD16: Cluster of differentiation 16; ICAM-1: Intercellular adhesion molecule-1; min.: minute; N/m: Newton per meter; nm: nanometer; μL: microliter; DLS: Dynamic light scattering; HBSS: Hank’s balanced salt solution; PBS: Phosphate buffered saline solution; MTC: Magnetic twisting cytometry; kHz: Kilohertz; Hz: Hertz; V: Volt.

## Competing interests

The author declares that there are no competing interests.

## Supplementary Material

Additional file 1: Table S1 Peak summary related to the hydrodynamic diameter distributions of TiO_2_ fine particles and nanoparticles. Click here for file

## References

[B1] Phillips L Barbano D The influence of fat substitutes based on protein and titanium dioxide on the sensory properties of low fat milk J Dairy Sci 1997 80 11 2726 10.3168/jds.S0022-0302(97)76234-9

[B2] Donachie M Titanium: A Technical Guide, in Titanium: A Technical Guide 1988 Metals Park Ohio: ASM International

[B3] Choudhary S Haberstroh K Webster T Enhanced functions of vascular cells on nanostructured Ti for improved stent applications Tissue Eng 2007 13 7 1421 1430 10.1089/ten.2006.0376 17518735

[B4] Branemark P-I Osseointegration and its experimental studies J Prosthetic Dentistry 1983 50 399 410 10.1016/S0022-3913(83)80101-2 6352924

[B5] Langford R Frame J Surface analysis of titanium maxillofacial plates and screws retrieved from patients Int J Oral Maxillofac Surg 2002 31 5 511 518 10.1054/ijom.2002.0283 12418567

[B6] Scales J Black staining around titanium alloy prostheses an orthopaedic enigma J Bone Joint Surg Br 1991 73 534 536 2071632 10.1302/0301-620X.73B4.2071632

[B7] Witt J Swann W Metal wear and tissue response in failed titanium alloy total hip replacements J Bone Joint Surg Br 1991 73 559 563 2071635 10.1302/0301-620X.73B4.2071635

[B8] Lygidakis N A retrieval study on morphological and chemical changes of titanium osteosynthesis plates and adjacent tissues J Cranio-Maxillofacial Surgery 2007 35 3 168 176 10.1016/j.jcms.2007.01.004 17583522

[B9] Case C Wide spread dissemination of metal debris from implants J Bone Joint Surg Br 1994 76 701 712 8083255

[B10] Sarmiento-González A Encinar JR Marchante-Gayón JM Sanz-Medel A Titanium levels in the organs and blood of rats with a titanium implant, in the absence of wear, as determined by double-focusing ICP-MS Anal Bioanal Chem 2009 393 1 335 343 10.1007/s00216-008-2449-2 18949464

[B11] Emsley J “Titanium”. Nature’s Building Blocks: An A-Z Guide to the Elements 2001 Oxford England, UK: Oxford University Press

[B12] Rosengren A Method for immunolocalization of extracellular proteins in association with the implant-soft tissue interface Biomaterials 1994 15 17 24 10.1016/0142-9612(94)90190-2 8161652

[B13] Aquino E Análise proteômica comparativa entre neutrófilos quiescentes e estimulados com fator de agregação plaquetária (PAF) 2008 Master Degree Dissertation. University of Brasilia, Biology Departament 149

[B14] Eriksson C Lausmaa J Nygren H Interactions between human whole blood and modified TiO_2_-surfaces: influence of surface topography and oxide thickness on leukocyte adhesion and activation Biomaterials 2001 22 14 1987 1996 10.1016/S0142-9612(00)00382-3 11426876

[B15] Tamura K Effects of micro/nano particle size on cell function and morphology Key Eng Mater 2004 254–256 919 922

[B16] Vamanu C Induction of cell death by TiO_2_ nanoparticles: studies on a human monoblastoid cell line Toxicol In Vitro 2008 22 1689 1696 10.1016/j.tiv.2008.07.002 18672048

[B17] Busch W Internalisation of engineered nanoparticles into mammalian cells in vitro: influence of cell type and particle properties J Nanoparticle Res 2011 13 1 293 310 10.1007/s11051-010-0030-3

[B18] Li N Ultrafine particulate pollutants induce oxidative stress and mitochondrial damage Environ Health Perspect 2003 111 455 460 12676598 10.1289/ehp.6000 PMC1241427

[B19] Geiser M Ultrafine particles cross cellular membranes by nonphagocytic mechanisms in lungs and in cultured cells Environ Health Perspect 2005 113 11 1555 1560 10.1289/ehp.8006 16263511 PMC1310918

[B20] Gonçalves DM Chiasson S Girard D Activation of human neutrophils by titanium dioxide (TiO2) nanoparticles Toxicol In Vitro 2010 24 3 1002 1008 10.1016/j.tiv.2009.12.007 20005940

[B21] Gonçalves DM Girard D Titanium dioxide (TiO2) nanoparticles induce neutrophil influx and local production of several pro-inflammatory mediators in vivo Int Immunopharmacol 2011 11 8 1109 1115 10.1016/j.intimp.2011.03.007 21426949

[B22] Torres M Coates T Function of the cytoskeleton in human neutrophils and methods for evaluation J Immunol Methods 1999 232 1–2 89 109 10618512 10.1016/s0022-1759(99)00168-4

[B23] Kumazawa R Effects of Ti ions and particles on neutrophil function and morphology Biomaterials 2002 23 3757 3764 10.1016/S0142-9612(02)00115-1 12109701

[B24] Okuda-Shimazaki J Effects of titanium dioxide nanoparticle aggregate size on gene expression Int J Mol Sci 2010 11 6 2383 2392 10.3390/ijms11062383 20640159 PMC2904923

[B25] Pereira J Estudo do Comportamento de nanopartículas de Dióxido de Titânio em diferentes suspensões 2010 Lisboa: Universidade de Lisboa 94

[B26] Kirchner C Cytotoxicity of colloidal CdSe and CdSe/ZnS nanoparticles Nano Lett 2005 5 2 331 338 10.1021/nl047996m 15794621

[B27] Grassian V Inflammatory response of mice to manufactured titanium dioxide nanoparticles: comparison of size effects through different exposure routes Nanotoxicology 2007 1 3 211 226 10.1080/17435390701694295

[B28] Arora H C.S.S.R. Kumar Nanomaterials for the Life Sciences in Nanocomposites 2010 Weinheim: WILEY-VCH Verlag GmbH & Co. KGaA

[B29] Zarbock A Ley K Mechanisms and consequences of neutrophil interaction with the endothelium Am J Pathol 2008 172 1 1 7 10.2353/ajpath.2008.070502 18079440 PMC2189633

[B30] Wang Q Doerschuk C The signaling pathways induced by neutrophil-endothelial cell adhesion Antioxid Redox Signal 2002 4 1 39 47 10.1089/152308602753625843 11970842

[B31] Witko-Sarsat V Rieu P Descamps-Latscha B Lesavre P Halbwachs-Mecarelli L Neutrophils: molecules, functions and pathophysiological aspects Lab Invest 2000 80 617 653 10.1038/labinvest.3780067 10830774

[B32] Long T Titanium dioxide (P25) produces reactive oxygen species in immortalized brain microglia (BV2): implications for nanoparticle neurotoxicity Environ Sci Technol 2006 40 14 4346 4352 10.1021/es060589n 16903269

[B33] Tan J Saltzman W Topographical control of human neutrophil motility on micropatterned materials with various surface chemistry Biomaterials 2002 23 3215 3225 10.1016/S0142-9612(02)00074-1 12102193

[B34] Eriksson C Nygren H Adhesion receptors of PMN granulocyteson titanium in contact with whole blood J Lab Clin Med 2001 137 56 63 10.1067/mlc.2001.111470 11150024

[B35] Watari F Material nanosizing effect on living organisms: non-specific, biointeractive, physical size effects J R Soc Interface 2009 6 S371 S388 10.1098/rsif.2008.0488.focus 19364724 PMC2690091

[B36] Kuznetsova T Atomic force microscopy probing of cell elasticity Micron 2007 38 8 824 833 10.1016/j.micron.2007.06.011 17709250

[B37] Vaziri A Kaazempur M Mechanics and deformation of the nucleus in micropipette aspiration experiment J Biomech 2007 40 2053 2062 10.1016/j.jbiomech.2006.09.023 17112531

[B38] Fabry B Time scale and other invariants of integrative mechanical behavior in living cells Phys Rev E 2003 68 041914 10.1103/PhysRevE.68.041914 14682980

[B39] Bhushan B Kawata S Hosaka S Bhushan B, Fuchs H, Harald Applied scanning probe methods I NanoScience and Technology P.A.B.B.D.B.K.v.H.S. R.Wiesendanger 2007 Berlin-Heidelberg: Springer-Verlag Berlin Heidelberg

[B40] Hayashi K Holzapfel GA Tensile Properties and Local Stiffness of Cells in Mechanics of Biological Tissue R.W.O 2006 Heidelberg: Springer Berlin 137 152

[B41] Acerbi I Integrin-specific mechanoresponses to compression and extension probed by cylindrical flat-ended AFM tips in lung cells PLoS One 2012 7 2 e32261 10.1371/journal.pone.0032261 22384196 PMC3285695

[B42] Seo Y Jhe W Atomic force microscopy and spectroscopy Rep Prog Phys 2008 71 1 016101 10.1088/0034-4885/71/1/016101

[B43] Butt H Cappella B Kappl M Force measurements with the atomic force microscope: Technique, interpretation and applications Surf Sci Rep 2005 59 1 152 10.1016/j.surfrep.2005.08.003

[B44] Neumann T JPK.Instruments Determining the elastic modulus of biological samples using atomic force microscopy 2009 AG, Berlin

[B45] Lee Y Patel D Park S Local rheology of human neutrophils investigated using atomic force microscopy Int J Biol Sci 2011 7 1 102 111 21278920 10.7150/ijbs.7.102 PMC3030146

[B46] Agrawal R Lee K Agrawal R Lee K Ponnavolu B AFM Spectroscopy, in Micro/Nano Science and Engineering 2004 Evanston- IL,USA: Department of Mechanical Engineering: Northwestern University

[B47] Hoh J Hansma P Atomic force microscopy for high-resolution imaging in cell biology Trends Cell Biol 1992 2 208 213 10.1016/0962-8924(92)90248-l 14731502

[B48] Rosenbluth M Lam W Fletcher D Force microscopy of nonadherent cells: a comparison of leukemia cell deformability Biophys J 2006 90 8 2994 3003 10.1529/biophysj.105.067496 16443660 PMC1414579

[B49] ASTM-F86-04 Standard Practice for Surface Preparation and Marking of metallic surgical Implants 2009 West Conshohocken, PA: Publisher-American Society for Testing and Materials

[B50] Teles L Comparison of the neutrophil proteome in trauma patients and normal controls Protein Pept Lett 2012 19 6 663 672 10.2174/092986612800493977 22519539 PMC3382372

[B51] Watari F Internal diffusion of micro/nanoparticles inside body Key Eng Mater 2007 361–363 95 98

[B52] Wang J Acute toxicity and biodistribution of different sized titanium dioxide particles in mice after oral administration Toxicol Lett 2007 168 176 185 10.1016/j.toxlet.2006.12.001 17197136

[B53] Johnston H Identification of the mechanisms that drive the toxicity of TiO_2_ particulates: the contribution of physicochemical characteristics Part Fibre Toxicol 2009 6 33 1 27 20017923 10.1186/1743-8977-6-33 PMC2804608

[B54] Bermudez E Pulmonary responses of mice, rats, and hamsters to subchronic inhalation of ultrafine titanium dioxide particles Toxicol Sci 2004 77 2 347 357 10.1093/toxsci/kfh019 14600271

[B55] Tamura K Effects of particle size on cell function and morphology in titanium and nickel Mater Trans 2002 43 12 3052 3057 10.2320/matertrans.43.3052

[B56] Guduru R In situ AFM Imaging of Nanoparticle- Cellular Membrane Interaction for a Drug Delivery Study 2011 Miami-Florida: FIU Electronic Thesis and Dissertations Paper 422

[B57] Smith B Cellular Biomechanics Investigated by Atomic Force Microscopy, in Centre for the Physics of Materials Department of Physics 2004 Montréal: McGill University

[B58] Hall W Single nanoparticle spectroscopy: an analysis of sample preparation and microscopy techniques Nanoscape 2005 2 1 35 41

[B59] Roca-Cusachs P Rheology of passive and adhesion-activated neutrophils probed by atomic force microscopy Biophys J 2006 91 9 3508 3518 10.1529/biophysj.106.088831 16891365 PMC1614490

[B60] Maddigan B Development of a model to explain the effect of variable membrane compliance on single molecule adhesive bond force 2004 Massachusetts USA: Massachusetts Institute of technology

[B61] Herant M Heinrich V Dembo M Mechanics of neutrophil phagocytosis: behavior of the cortical tension J Cell Sci 2005 1 118(Pt 9) 1789 1797 15827090 10.1242/jcs.02275

[B62] Kendall K “Adhesion: molecules and mechanics” Science 1994 263 5154 1720 1725 10.1126/science.263.5154.1720 17795378

[B63] Barbosa E Silva LP Nanoscale characterization of synthetic polymeric porous membranes: Scrutinizing their stiffness, roughness, and chemical composition J Membr Sci 2012 407–408 128 135

[B64] Roca-Cusachs P Neutrophil microrheology probed by atomic force microscopy FASEB J 2006 20 A 1296

[B65] Stomp R Dissipative and Electrostatic Force Spectroscopy of InAs Quantum Dots by Non-contact Atomic Force Microscopy, in Centre for the Physics of Materials Department of Physics McGill University Montrèal 2005 Quebec- Canada: McGill University 131

[B66] Vorden D Lange M Moller R Energy dissipation in dynamic force spectroscopy of PTCDA on Ag-Si(111) √3 × √3 e-J Surf Sci Nanotech 2011 9 21 25

[B67] Fok TNA Tang J Imaging and Stiffness Probing of Neutrophil Using Atomic Force Microscope 2008 Hong Kong: Department of Physics, The Chinese University of Hong Kong Physics Department, Brown University, Providence, RI

[B68] Ananthakrishnan R Ehrlicher A The forces behind cell movement Int J Biol Sci 2007 3 5 303 317 17589565 10.7150/ijbs.3.303 PMC1893118

