# Supplementary material for: Kinetic effects of TiO2 fine particles and nanoparticles aggregates on the nanomechanical properties of human neutrophils assessed by force spectroscopy
Source: BMC Biophys. 2013 Aug 19;6:11. doi: 10.1186/2046-1682-6-11 (PMC3766645; doi:10.1186/2046-1682-6-11)
Supplement: Additional file 1: Table S1 — Peak summary related to the hydrodynamic diameter distributions of TiO2 fine particles and nanoparticles. [file 2046-1682-6-11-S1.doc]

Additional file 1: Table S1. Peak summary related to the hydrodynamic diameter distributions of TiO_2_ fine particles and nanoparticles

| **Diameter (nm)** | **Volume (%)** | **Width** |
| --- | --- | --- |
| **180.9** | 67.6 | 151.40 |
| **9.00** | 22.1 | 4.66 |
| **0.980** | 10.3 | 0.22 |
